# Sequential sampling models with variable boundaries and non-normal noise: A comparison of six models

**DOI:** 10.3758/s13423-018-1560-4

**Published:** 2019-01-16

**Authors:** Andreas Voss, Veronika Lerche, Ulf Mertens, Jochen Voss

**Affiliations:** 1 Institute of Psychology, Heidelberg University, Hauptstrasse 47-51, D-69117 Heidelberg, Germany; 2 School of Mathematics, University of Leeds, Leeds, UK

**Keywords:** Diffusion model, Lévy flight model, Collapsing boundaries, Decision making

## Abstract

One of the most prominent response-time models in cognitive psychology is the diffusion model, which assumes that decision-making is based on a continuous evidence accumulation described by a Wiener diffusion process. In the present paper, we examine two basic assumptions of standard diffusion model analyses. Firstly, we address the question of whether participants adjust their decision thresholds during the decision process. Secondly, we investigate whether so-called Lévy-flights that allow for random jumps in the decision process account better for experimental data than do diffusion models. Specifically, we compare the fit of six different versions of accumulator models to data from four conditions of a number-letter classification task. The experiment comprised a simple single-stimulus task and a more difficult multiple-stimulus task that were both administered under speed versus accuracy conditions. Across the four experimental conditions, we found little evidence for a collapsing of decision boundaries. However, our results suggest that the Lévy-flight model with heavy-tailed noise distributions (i.e., allowing for jumps in the accumulation process) fits data better than the Wiener diffusion model.

## Introduction

The diffusion model was introduced four decades ago as a tool to analyze response-time data (Ratcliff, 1978). However, only in the last two decades has the model become widely popular in cognitive psychology (Voss, Nagler, & Lerche, 2013). There are many reasons for this recent increase in popularity: Firstly, the diffusion model provides good fit to data from a wide variety of cognitive tasks, and the model’s parameters have been validated successfully for different paradigms (e.g., Arnold, Broder, & Bayen, 2015; Lerche & Voss, 2017; Voss, Rothermund, & Voss, 2004). Thus, there is evidence that the diffusion model reflects the true processes in fast binary decisions reasonably well and that it allows for disentangling these processes. Secondly, the diffusion model proved to be a useful tool to test specific psychological hypotheses. Most importantly, it solves a central problem of measuring cognitive performance with response-time tasks: In typical experimental tasks, performance spreads over two metrics, i.e., response latencies and accuracy rates. Traditional analyses often use either mean response times (typically from correct responses only) or accuracy. This is obviously problematic: If – on the one hand – results from speed and accuracy analyses point in the same direction (i.e., responses in one condition are faster and more accurate compared to the other condition), the spreading of the effect of condition on the two metrics might reduce the power to detect the effect. If – on the other hand – conditions have opposite effects on latencies and accuracy, wrong conclusions might be drawn, because the observed effects reflect a change in response style rather than in performance. A diffusion-model analysis solves this problem by providing independent measures for performance and for the adopted speed-accuracy setting (Spaniol, Madden, & Voss, 2006). The model also allows defining an optimal decision-making strategy in terms of speed-accuracy settings (Bogacz, Brown, Moehlis, Holmes, & Cohen, 2006). A third reason for the popularity of the diffusion model is the strong assocation of the model's architecture with neural processes (Gold & Shadlen, 2007).

Despite these advantages of diffusion modelling, its application was initially limited by the difficulties of implementing this approach. As often is the case in mathematical psychology, the lack of available software and of accessible tutorials is a critical barrier that prevents the spreading of innovative models. About 10 years ago, however, the development of user-friendly software tools finally allowed many researchers to use this powerful method (Grasman, Wagenmakers, & van der Maas, 2009; Vandekerckhove & Tuerlinckx, 2008; Voss & Voss, 2007; Voss, Voss, & Lerche, 2015; Wagenmakers, van der Maas, & Grasman, 2007; Wiecki, Sofer, & Frank, 2013).

While a diffusion-model analysis may seem mathematically and computationally challenging, it still is a rather simple model of decision-making. In the present research, we wanted to challenge some of the simplifying assumptions made by the standard diffusion model. Specifically, the assumptions of constant thresholds and of Gaussian noise are addressed here. To do this, we compare the model fit of different variants of accumulator models. We discuss reasons for possible violations of these assumptions – and consequences of not accounting for them in models – after briefly introducing the standard diffusion model (for a more thorough introduction, see Ratcliff & McKoon, 2008; Voss et al., 2013).

### The standard diffusion model

In diffusion-model analyses, it is assumed that binary decisions are based on a continuous accumulation of information. Evidence accumulation starts at point *z* on a dimension representing subjective support for the two possible decisional outcomes, and it moves over time – depending on the perceived information – upwards or downwards with a mean slope *v* until it reaches a lower bound (at 0) or an upper bound (at *a*). This information accumulation is assumed to be noisy; that is, to a constant time-dependent change in subjective evidence is added Gaussian noise. Thus, a Wiener diffusion process running in a corridor between two thresholds describes information accumulation. First passage times represent decision latencies and the thresholds that are hit by the process denote the decisional outcomes.

The parameters describing this model have been shown to map specific psychological aspects of the decision process (Voss et al., 2004): The mean slope of the process, *drift v*, is a measure of the speed of information entering the decision process and can be taken as a measure of task difficulty (in the comparison of tasks) or of cognitive speed (in the comparison of individuals). *Threshold separation* (*a*) represents the speed-accuracy setting of a decision-maker: Small distances indicate a focus on speed (fast but error-prone decisions), whereas large threshold separations suggest a focus on accuracy (accurate but slow decisions). The *start point* (*z*) maps decisional biases: For an unbiased decision-maker, information accumulation will start centered between thresholds. If, however, the decision-maker expects – or just hopes for – a specific response to be correct in the upcoming decision, the start point will be positioned closer to the corresponding threshold. Further, the duration of *non-decisional processes* (*t*_*0*_; time of stimulus encoding and motor processes) needs to be included in the model. This is done by adding *t*_*0*_ to the decision times predicted by the diffusion process.

Additional parameters are often included in the model to account for inter-trial variability of drift, start point, and non-decision time (Ratcliff & Rouder, 1998; Ratcliff & Tuerlinckx, 2002). For the complete diffusion model, it is assumed that drift across trials follows a normal distribution with mean *v* and standard deviation *s*_*v*_. For start point and non-decision time, uniform distributions with means *z* and *t*_0_ and widths *s*_*z*_ and *s*_*t*0_ are used. Previous research showed that – in the case of small trial numbers – estimation can be more stable without these inter-trial variability parameters (Lerche & Voss, 2016). We therefore decided to fit both the simple diffusion model (without between-trial dynamics) as well as the full diffusion model to our data.

In the remainder of this paper, we refer to the standard diffusion model with Gaussian noise as the “Fixed boundary model” (Model 1), because in this model decision thresholds are assumed to be constant over time.

### Changing from conservative to liberal: models with dynamic adaptation of thresholds

In the past, the diffusion model was often applied to data from fast perceptual decisions; Roger Ratcliff often recommended using the model exclusively for tasks with mean latencies below 1.5 s (e.g., Ratcliff & McKoon, 2008). However, recent results from our own lab suggest that the diffusion model might also be useful for tasks with notably slower responses (Lerche & Voss, 2017). One challenge of applying the model to slow decision tasks is the postulation of constant thresholds. Typically, it is assumed that the upper threshold remains at *a* and the lower threshold at zero, no matter how long the information accumulation takes. However, if the task is very difficult, that is, if drift is close to zero, it might take a while before sufficient information accumulates.^1^ In this case, it becomes more and more plausible that decision-makers change response caution within one trial and eventually start collapsing decision boundaries. First studies testing collapsing boundary models for fast decision tasks seem to support the assumption of constant thresholds (Hawkins, Forstmann, Wagenmakers, Ratcliff, & Brown, 2015). In the present research, we apply different collapsing boundary models to an easier and a more difficult task. As argued above, we consider a dynamic adaptation of thresholds more likely for the more difficult task.

**Fig. 1:**
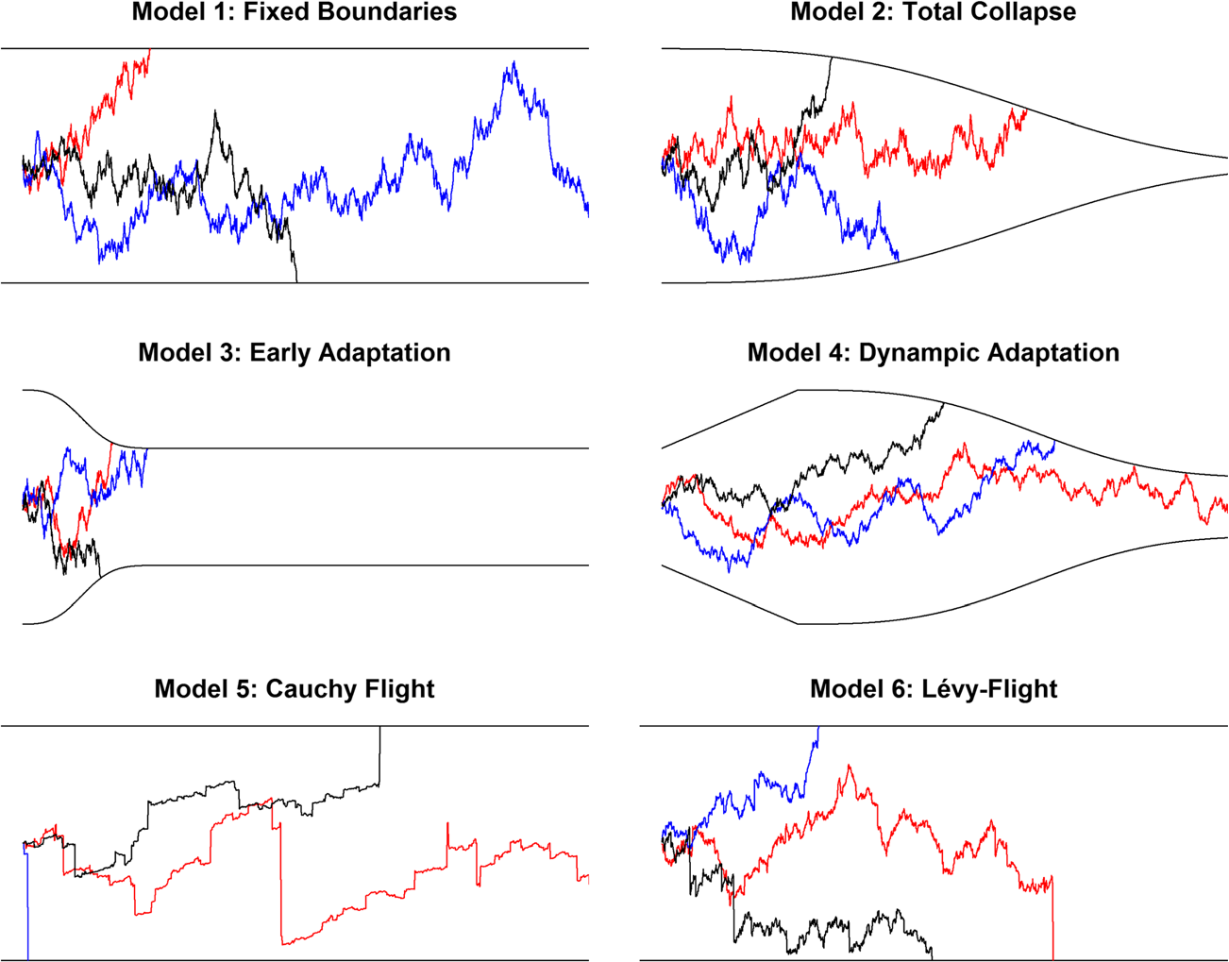
Schematic representation of the six decision models with three sample paths for evidence accumulation. Further explanations are given in the text

We examine three forms of dynamic adaptation of thresholds (Fig. 1, Models 2–4; see also Table 1): The first of these models (Model 2: Total Collapse) assumes a total collapse of thresholds, that is, with increasing decision times, the distance between the upper and lower thresholds approaches zero. Following Hawkins et al. (2015), we model the collapse of boundaries with a Weibull cumulative distribution function. Equation (1) provides the upper threshold *u* at time *t* (*t* refers to decision time only, i.e., it excludes non-decision time).

1 $$ u(t)=a-\left(1-\exp \left(-{\left(\frac{t}{\lambda}\right)}^k\right)\right)\cdotp \left(-0.5\cdotp \delta \cdotp a\right) $$

**Table 1.** Model variants

Model	Free parameters	Fixed parameters
1. Fixed Boundaries	*a*, *v*_*easy*_, (*v*_*diff*_), *t*_*0,*_ (*s*_*ν*_, *s*_*zr*_, *s*_*t0*_)	*α* = 2
2. Total Collapse	*a*, *v*_*easy*_, (*v*_*diff*_), *t*_*0*_, *λ*, (*s*_*ν*_, *s*_*zr*_, *s*_*t0*_)	*α* = 2, *δ* = − 1
3. Early Adaptation	*a*, *v*_*easy*_, (*v*_*diff*_), *t*_*0*_, *δ*, (*s*_*ν*_, *s*_*zr*_, *s*_*t0*_)	*α* = 2, *λ* = 0.25
4. Dynamic Adaptation	*a*, *v*_*easy*_, (*v*_*diff*_), *t*_*0*_*, δ, λ,* (*s*_*ν*_, *s*_*zr*_, *s*_*t0*_)	*α* = 2
5. Cauchy Flight	*a*, *v*_*easy*_, (*v*_*diff*_), *t*_*0*_*,* (*s*_*ν*_, *s*_*zr*_, *s*_*t0*_)	*α* = 1
6. Lévy Flight	*a*, *v*_*easy*_, (*v*_*diff*_), *t*_*0*_*, α,* (*s*_*ν*_, *s*_*zr*_, *s*_*t0*_)	

Notes: *a*=threshold separation; *v*=drift; *t*_*0*_=non-decision time; *α*=stability parameter of stable distribution (normal distribution: *α* = 2; Cauchy distribution: *α* = 1); *λ*=onset of collapse; *δ*=amount of collapse (-1=total collapse). For the single-stimulus conditions, only one drift rate was estimated; for the multi-stimulus conditions, two drift rates were estimated. For all models, relative starting point (*z*/*a*) was set at *z*_*r*_ = .5. All six model were fitted to data with and without including inter-trial variabilities (s_v_, s_zr_. s_t0_) as three additional free parameters

In this equation, *k* governs the shape of collapse (early vs. late), *λ* (the scale parameter of the Weibull distribution) determines the onset at which the collapse begins, *δ* is the amount of collapse (with *δ* =  − 1 indicating a total collapse) and *a* denotes initial threshold separation. For the *total collapse model*, shape and amount of collapse are fixed at *k* = 3 and *δ* =  − 1, and *λ* is a free parameter. These parameter settings were used by Hawkins et al. (2015) to model a "late collapse decision strategy." The lower threshold (*l*) is assumed to be symmetrical and was calculated as:

2 $$ l(t)=a-u(t) $$

In a second model with dynamic boundaries (Model 3: Early Adaptation), an early adaption of thresholds is assumed. This is done by setting the onset of collapse in Eq. (1) to *λ* = 0.25 s, and estimating the amount of collapse (*δ*) as free parameter. Note that this model also allows for an early increase in thresholds, which would be indicated by *δ* > 0. The choice of 250 ms for lambda is based on the response times expected in our tasks (see below). A reasonable value for lambda needs to allow for a notable adaption of thresholds before the majority of decisions are completed.

A third model with dynamic thresholds (Model 4: Dynamic Adaptation) combines the possibility of an early adaptation of thresholds with later total collapse. We included this model because data from a pilot study suggested early increases in threshold distances for a substantial number of participants. For this model, we assume a linear adaptation of thresholds in the first 500 ms, followed by a Weibull shaped total collapse:

3 $$ u(t)=\left\{\begin{array}{l}a+\frac{t}{0.5}\delta, \kern0.5em \mathrm{for}\ t\le 0.5\\ {}\left(a+{a}^{\prime}\right)-\left(1-\exp \left(-{\left(\frac{\left(t-0.5\right)}{\lambda}\right)}^k\right)\right)\cdotp \left(0.5\cdotp \left(a+\delta \right)\right),\kern0.5em \mathrm{for}\ t>0.5\kern1em \end{array}\right. $$

In this model, *a*^′^ indicates the adaptation after a linear change in thresholds for the first 500 ms. The amount of the Weibull-shaped total collapse was already set to −1 in Eq. (3). Thus, the shape of thresholds in Model 4 is determined by the free parameters *a*, *δ* and *λ*.

### Jumping to conclusions: Lévy-flight models with non-normal noise

In diffusion models, the noise of information accumulation follows a Gaussian distribution. Lévy-flights, in contrast, allow for so-called heavy-tailed noise distribution, like – for example – the Lévy or the Cauchy distribution.^2^ Heavy tails of the noise distribution allow for large jumps in the modelled process, and thus make it possible to incorporate extreme events, like a crash at the stock market (Mantegna, 1991) or sudden large changes in the hunting area of predators like sharks (Raichlen et al., 2014). We believe that such sudden changes in subjective decision strength can also occur in human decision-making, suggesting that Lévy-flight models might account better for the real cognitive processes in binary decision tasks than diffusion models do. In Model 5 (Cauchy-flight), we assume noise to follow a Cauchy distribution. Both normal distribution and Cauchy distribution are special cases of the broader class of so-called Lévy alpha-stable distributions. The heaviness in the tails of the distribution (i.e., the probability of extreme events) is controlled by the stability parameter α, with a normal distribution and Cauchy distribution characterized by *α* = 2.0 or *α* = 1.0, respectively. In Model 6 (Lévy-flight^3^), alpha is not fixed but estimated as a free parameter.

### Fitting the models to data

Unlike for the diffusion model, for which density functions and cumulative probability functions (CDF) are known (Blurton, Kesselmeier, & Gondan, 2012; Navarro & Fuss, 2009; Voss & Voss, 2008; Voss et al., 2015), the calculation of predicted response dime distributions is much more difficult for the more complex collapsing-boundary models or Lévy-flight models. In the fitting procedure, we approximate the density by simulating many paths of the decision process with a given model class and given parameters. The diffusion process (Models 1–4) is simulated by the stochastic Euler method following Eq. (4),

4 $$ x\left(t+\Delta t\right)=x(t)+\nu \cdotp \Delta t+e\sqrt{\Delta t},\mathrm{with}\ e\sim N\left(0,1\right), $$

where *t* is the time, Δ*t* is a small time increment (1 ms in our simulations), *v* is the drift of the diffusion process, and *e* is normally distributed noise.^4^ The process starts at *x*(0) = *z*, and runs until it exceeds the upper or lower thresholds, which is constant for Model 1 and is adapted as a function of time in Models 2–4.

For a Cauchy-flight (Model 5) and – more generally – for a Lévy-flight (Model 6) assuming stable-distributed noise, the path is calculated as:

5 $$ x\left(t+\Delta t\right)=x(t)+\nu \cdotp \Delta t+e\Delta t,\mathrm{with}\ e\sim Cauchy\left(0,1\right),\mathrm{or} $$

6 $$ x\left(t+\Delta t\right)=x(t)+\nu \cdotp \Delta t+e\Delta {t}^{1/\alpha },\mathrm{with}\ e\sim Stable\left(\alpha, \beta =0,\gamma =1,\delta =0\right), $$

respectively. Note that Eqs. (4) and (5) are special cases of Eq. (6) with *α* = 2 for the normal distribution, and *α* = 1 for the Cauchy distribution, respectively.

Models were fitted using quantile maximum probability estimation (Brown & Heathcote, 2003; Heathcote & Brown, 2004). This procedure is based on the massive simulation of data from a given set of parameter values. Probabilities for observed responses are then estimated from the relative frequency of simulated responses falling into predefined response-time bins for correct and error responses. Following the logic of a log-likelihood approach, a search for a set of parameters is performed that maximizes the sum of logarithmized response probabilities. For this purpose we used the SIMPLEX algorithm (Nelder & Mead, 1965).

Parameter estimation was implemented as a C program running on a large computer cluster. Random-number generation and the SIMPLEX search used the implementations from the GNU Scientific Library.^5^ Quantile probabilities were estimated based on 20,000 simulated responses for each step of the search procedure. For the easy task (see below), probabilities were estimated for response-time bins ranging from 300 ms to 3,000 ms, with a bin size of 20 ms. For the difficult task, bins had a width of 50 ms and ranged from 300 ms to 10,000 ms. After the search converged, the likelihood of the resulting parameter set was re-assessed with a higher accuracy drawing on 200,000 simulated responses. A simulation study demonstrating the quality of parameter recovery is presented in Appendix A.

## Experiment

### Method

#### Participants

The sample consisted of 81 participants (63 females; mean age = 24 years; range: 17–24) who were recruited from an online participants’ database at Heidelberg University using the software hroot (Bock, Baetge, & Nicklisch, 2014). Most of them were undergraduate students with varying majors. All participants gave informed consent prior to the experiment and were compensated for their participation with 8 Euro or partial course credit.

#### Design

The design consisted of the within-participant factors *task* (single-stimulus vs. multi-stimulus) and *instruction* (speed vs. accuracy). In the multi-stimulus conditions, the task *difficulty* also varied between trials (easy vs. difficult).

#### Procedure

The experiment was conducted in group sessions with up to 25 participants working simultaneously. Participants completed four experimental blocks in a random sequence. Each block started with the presentation of instructions, which either stressed the importance of accurate or of fast responding. Each block comprised eight practice trials followed by 160 experimental trials that were presented in an individually randomized order. Each trial started with the presentation of a fixation cross for 500 ms. In the single-stimulus task, the fixation cross was then replaced by either a number (1–9) or a letter (A–I), both presented in Times New Roman font (size: 48 point). The stimulus remained on screen until a response was given with the A-key (for numbers) or the L-key (for letters) on a standard keyboard. Labels for the assignment of keys were present at the bottom corners of the screen throughout the experiment. Only in the eight practice trials of each block was accuracy feedback provided. Following the response, the next trial started after a black screen of 500 ms.

In the multi-stimulus blocks, the sequence of a trial was similar. However, instead of one target stimulus, a total of 16 letters and numbers were presented simultaneously. The task was now to decide whether more letters or more numbers were shown. In easy trials, there were four letters and 12 numbers or vice versa. In difficult trials, there were six letters and ten numbers or vice versa. To prevent exact counting, the stimuli moved in random directions across the complete screen.^6^ In the multi-stimulus condition, inter-trial intervals were 1,500 ms.

### Results

#### Data pre-treatment

Data from trials with latencies below 100 ms (0.08% of all trials) and above 20 s (0.05% of all trials) were removed prior to all analyses.

#### Response times and accuracy

Mean response times (RTs) and accuracy values for all conditions are presented in Table 2. A 2 (task: single-stimulus vs. multi-stimulus) × 2 (instruction: speed vs. accuracy) × 2 (accuracy: correct vs. error responses) repeated measurement ANOVA^7^ of mean RTs revealed substantial main effects of task, *F*(1,68)=166.53, *p*<.001, ${\eta}_G^2$=.44, instruction, *F*(1,68)=53.72, *p*<.001, ${\eta}_G^2$=.09, and accuracy, *F*(1,68)=9.10, *p*=.004, ${\eta}_G^2$=.01, indicating slower responses for the multiple stimulus condition, for accuracy instructions, and – overall – also for error responses. These main effects were qualified by a row of interactions: Firstly, the effect of instruction on RT was more pronounced in the multi-stimulus task, *F*(1,68)=48.44, *p*<.001, ${\eta}_G^2$=.07. Secondly, the effect of accuracy on mean response time depended on task, *F*(1,68)=20.26, *p*<.001, ${\eta}_G^2$=.01, with fast errors in the single-stimulus condition and slow errors in the multiple-stimulus condition. Thirdly, the main effect of accuracy on RT was slightly increased after accuracy instruction, *F*(1,68)=4.46, *p*=.04, ${\eta}_G^2$=.001. Finally, a three-way interaction suggests that this instruction by accuracy interaction is present only in the multi-stimulus task, *F*(1,68)=4.70, *p*=.03, ${\eta}_G^2$=.001.

**Table 2:** Mean response times and accuracies (standard deviations in parentheses) for the four conditions of the letter-number experiment

Task	Single-stimulus task		Multi-stimulus task	
Instruction	Speed	Accuracy	Speed	Accuracy
Response times (correct)	444 (58)	502 (89)	1,110 (420)	1,847 (921)
Response times (error)	397 (73)	445 (98)	1,258 (617)	2,225 (1,558)
Accuracy	.92 (.06)	.96 (.04)	.83 (.08)	.89 (.07)

Similar effects were found for accuracy rate, which was lower in the multi-stimulus task, *F*(1,80)=163.65, *p*<.001, ${\eta}_G^2$=.29, and after speed instructions, *F*(1,80)=92.71, *p*<.001, ${\eta}_G^2$=.14. Again, the effect of instruction was increased for data from the multi-stimulus task, *F*(1,80)=12.41, *p*<.001, ${\eta}_G^2$=.01.

#### Parameter estimates

Parameters were estimated separately for each model, each condition, and each participant. Mean parameter values for all six models are presented in Table 3. For each model each parameter was analyzed with a 2 (Task) × 2 (Instruction) repeated measurement ANOVA.^8^ Table 4 shows the effect sizes (generalized η^2^) for all analyses. As expected, all models detected an effect of speed versus accuracy instructions on (initial) threshold separation. Less information was required for a response under speed instructions compared to accuracy instructions, all *Fs*(1,80)≥65.29; *ps*<.001. Additionally, threshold separation was generally increased for the more difficult multi-stimulus task, all *Fs*(1,80)≥37.48; *ps*<.001.

**Table 3:** Mean parameter values (SDs in parentheses)

Task	*a*	*v*_*easy*_	*v*_*diff*_	*t*_0_	*s*_*zr*_	*s*_*v*_	*st*_0_	*α*	*λ*	*δ*
Model 1: Standard Diffusion (simple)										
Single-speed	1.19 (0.30)	3.41 (1.12)	---	0.27 (0.03)	---	---	---	---	---	---
Single-acc	1.62 (0.69)	3.74 (1.28)	---	0.28 (0.04)	---	---	---	---	---	---
Multi-speed	1.90 (0.56)	1.40 (0.47)	0.78 (0.36)	0.40 (0.10)	---	---	---	---	---	---
Multi-acc	2.79 (1.02)	1.26 (0.39)	0.66 (0.25)	0.40 (0.15)	---	---	---	---	---	---
Model 2: Total Collapse (simple)										
Single-speed	1.11 (0.29)	3.04 (0.97)	---	0.27 (0.03)	---	---	---	---	1.39 (0.34)	---
Single-acc	1.57 (0.74)	3.52 (1.28)	---	0.28 (0.04)	---	---	---	---	1.41 (0.39)	---
Multi-speed	2.04 (0.75)	1.35 (0.40)	0.68 (0.23)	0.37 (0.11)	---	---	---	---	2.83 (1.08)	---
Multi-acc	3.07 (1.13)	1.24 (0.40)	0.64 (0.19)	0.33 (0.16)	---	---	---	---	4.61 (2.52)	---
Model 3: Early Adaptation (simple)										
Single-speed	1.14 (0.30)	3.06 (0.99)	---	0.27 (0.03)	---	---	---	---	---	-0.10 (0.23)
Single-acc	1.60 (0.74)	3.69 (1.32)	---	0.28 (0.04)	---	---	---	---	---	-0.04 (0.19)
Multi-speed	1.90 (0.53)	1.40 (0.42)	0.73 (0.25)	0.40 (0.10)	---	---	---	---	---	-0.02 (0.11)
Multi-acc	2.88 (1.08)	1.32 (0.38)	0.69 (0.21)	0.40 (0.15)	---	---	---	---	---	0.01 (0.10)
Model 4: Dynamic Adaptation (simple)										
Single-speed	1.17 (0.30)	3.03 (1.00)	---	0.27 (0.03)	---	---	---	---	1.49 (0.57)	-0.11 (0.18)
Single-acc	1.59 (0.72)	3.52 (1.32)	---	0.28 (0.04)	---	---	---	---	1.41 (0.39)	-0.07 (0.17)
Multi-speed	2.04 (0.72)	1.33 (0.40)	0.71 (0.26)	0.38 (0.10)	---	---	---	---	2.77 (1.08)	-0.07 (0.18)
Multi-acc	3.07 (1.38)	1.26 (0.39)	0.65 (0.22)	0.35 (0.17)	---	---	---	---	4.29 (2.43)	-0.03 (0.34)
Model 5: Cauchy-flight (simple)										
Single-speed	1.36 (0.57)	2.89 (1.01)	---	0.21 (0.05)	---	---	---	---	---	---
Single-acc	1.84 (0.77)	3.39 (1.13)	---	0.21 (0.05)	---	---	---	---	---	---
Multi-speed	1.68 (0.78)	1.27 (0.41)	0.82 (0.34)	0.43 (0.11)	---	---	---	---	---	---
Multi-acc	2.97 (1.57)	1.31 (0.38)	0.80 (0.28)	0.47 (0.16)	---	---	---	---	---	---
Model 6: Lévy-flight (simple)										
Single-speed	1.20 (0.47)	3.15 (1.25)	---	0.25 (0.04)	---	---	---	1.51 (0.29)	---	---
Single-acc	1.68 (0.74)	3.76 (1.33)	---	0.27 (0.04)	---	---	---	1.71 (0.24)	---	---
Multi-speed	1.81 (0.69)	1.35 (0.39)	0.74 (0.26)	0.42 (0.10)	---	---	---	1.71 (0.32)	---	---
Multi-acc	2.89 (1.21)	1.33 (0.36)	0.71 (0.21)	0.43 (0.16)	---	---	---	1.82 (0.26)	---	---
Model 1: Standard diffusion (full)										
Single-speed	1.03 (0.27)	4.39 (0.84)	---	0.32 (0.03)	0.64 (0.15)	1.22 (0.43)	0.12 (0.06)	---	---	---
Single-acc	1.31 (0.33)	4.50 (0.88)	---	0.34 (0.04)	0.49 (0.18)	1.09 (0.36)	0.11 (0.06)	---	---	---
Multi-speed	1.95 (0.70)	1.85 (0.51)	0.98 (0.31)	0.49 (0.09)	0.42 (0.18)	0.91 (0.48)	0.27 (0.12)	---	---	---
Multi-acc	2.91 (1.02)	1.59 (0.51)	0.83 (0.26)	0.50 (0.09)	0.36 (0.15)	0.51 (0.36)	0.30 (0.14)	---	---	---
Model 2: Total collapse (full)										
Single-speed	1.02 (0.27)	4.43 (0.87)	---	0.32 (0.03)	0.66 (0.15)	1.29 (0.48)	0.12 (0.05)	---	1.04 (0.04)	---
Single-acc	1.31 (0.34)	4.54 (0.91)	---	0.34 (0.04)	0.51 (0.20)	1.19 (0.40)	0.11 (0.06)	---	1.25 (0.26)	---
Multi-speed	2.03 (0.71)	1.85 (0.59)	0.95 (0.34)	0.51 (0.20)	0.43 (0.20)	0.92 (0.56)	0.35 (0.39)	---	2.09 (0.29)	---
Multi-acc	2.99 (1.06)	1.52 (0.53)	0.77 (0.30)	0.45 (0.15)	0.28 (0.18)	0.51 (0.41)	0.24 (0.17)	---	4.02 (1.83)	---
Model 3: Early adaptation (full)										
Single-speed	1.02 (0.27)	4.47 (0.88)	---	0.32 (0.03)	0.66 (0.16)	1.36 (0.45)	0.12 (0.05)	---	---	0.02 (0.09)
Single-acc	1.31 (0.34)	4.64 (0.88)	---	0.34 (0.04)	0.52 (0.19)	1.21 (0.43)	0.11 (0.06)	---	---	0.00 (0.09)
Multi-speed	1.97 (0.71)	1.92 (0.53)	1.00 (0.30)	0.50 (0.10)	0.44 (0.18)	0.97 (0.55)	0.32 (0.15)	---	---	-0.02 (0.06)
Multi-acc	2.95 (1.07)	1.63 (0.52)	0.86 (0.25)	0.52 (0.10)	0.39 (0.17)	0.54 (0.39)	0.32 (0.15)	---	---	0.00 (0.05)
Model 4: Dynamic adaptation (full)										
Single-speed	1.02 (0.27)	4.47 (0.87)	---	0.32 (0.03)	0.66 (0.15)	1.30 (0.43)	0.12 (0.05)	---	1.04 (0.03)	-0.01 (0.08)
Single-acc	1.30 (0.34)	4.58 (0.89)	---	0.34 (0.04)	0.51 (0.20)	1.17 (0.44)	0.11 (0.06)	---	1.18 (0.20)	0.00 (0.08)
Multi-speed	1.99 (0.77)	1.89 (0.54)	0.97 (0.29)	0.51 (0.13)	0.42 (0.19)	0.92 (0.53)	0.33 (0.30)	---	2.05 (0.08)	-0.02 (0.12)
Multi-acc	3.01 (1.28)	1.56 (0.55)	0.82 (0.28)	0.45 (0.14)	0.30 (0.17)	0.51 (0.39)	0.26 (0.15)	---	3.70 (1.87)	0.01 (0.21)
Model 5: Cauchy-flight (full)										
Single-speed	1.05 (0.26)	4.52 (0.78)	---	0.31 (0.03)	0.67 (0.14)	1.48 (0.42)	0.11 (0.05)	---	---	---
Single-acc	1.32 (0.33)	4.60 (0.76)	---	0.33 (0.04)	0.55 (0.18)	1.36 (0.34)	0.11 (0.06)	---	---	---
Multi-speed	1.87 (0.82)	1.93 (0.52)	1.09 (0.33)	0.50 (0.10)	0.49 (0.21)	1.07 (0.67)	0.27 (0.15)	---	---	---
Multi-acc	3.04 (1.27)	1.64 (0.48)	0.93 (0.31)	0.54 (0.11)	0.41 (0.20)	0.56 (0.47)	0.27 (0.17)	---	---	---
Model 6: Lévy-flight (full)										
Single-speed	1.06 (0.28)	4.56 (0.86)	---	0.32 (0.03)	0.66 (0.15)	1.42 (0.44)	0.12 (0.05)	1.58 (0.46)	---	---
Single-acc	1.34 (0.34)	4.71 (0.83)	---	0.33 (0.04)	0.52 (0.19)	1.30 (0.37)	0.11 (0.06)	1.53 (0.43)	---	---
Multi-speed	1.92 (0.76)	1.97 (0.50)	1.03 (0.29)	0.51 (0.10)	0.47 (0.19)	0.99 (0.58)	0.30 (0.14)	1.73 (0.36)	---	---
Multi-acc	2.99 (1.13)	1.66 (0.50)	0.91 (0.26)	0.53 (0.11)	0.39 (0.19)	0.55 (0.39)	0.31 (0.16)	1.82 (0.28)	---	---

**Table 4:** Effects of the treatment (generalized η^2^) on estimated parameters

Effect	*a*	*v*_*easy*_	*v*_*diff*_	*t*_0_	*s*_*zr*_	*s*_*v*_	*st*_0_	*α*	*λ*	*δ*
Model 1: Standard diffusion (simple)										
Task	0.318	0.609	---	0.335	---	---	---	---	---	---
Instruction	0.185	0.003	0.040	0.001	---	---	---	---	---	---
T x I	0.027	0.016	---	0.001	---	---	---	---	---	---
Model 2: Total collapse (simple)										
Task	0.376	0.580	---	0.143	---	---	---	---	0.413	---
Instruction	0.185	0.012	0.010	0.007	---	---	---	---	0.096	---
T x I	0.032	0.028	---	0.013	---	---	---	---	0.091	---
Model 3: Early adaptation (simple)										
Task	0.336	0.576	---	0.326	---	---	---	---	---	0.038
Instruction	0.200	0.025	0.007	0.001	---	---	---	---	---	0.020
T x I	0.031	0.041	---	0.001	---	---	---	---	---	0.002
Model 4: Dynamic adaptation (simple)										
Task	0.316	0.566	---	0.182	---	---	---	---	0.367	0.007
Instruction	0.148	0.014	0.015	0.002	---	---	---	---	0.065	0.007
T x I	0.030	0.025	---	0.012	---	---	---	---	0.080	0.000
Model 5: Cauchy-flight (simple)										
Task	0.119	0.571	---	0.577	---	---	---	---	---	---
Instruction	0.168	0.028	0.001	0.013	---	---	---	---	---	---
T x I	0.040	0.021	---	0.009	---	---	---	---	---	---
Model 6: Lévy-flight (simple)										
Task	0.237	0.557	---	0.408	---	---	---	0.074	---	---
Instruction	0.187	0.024	0.005	0.007	---	---	---	0.071	---	---
T x I	0.033	0.027	---	0.000	---	---	---	0.008	---	---
Model 1: Standard diffusion (full)										
Task	0.485	0.790	---	0.582	0.227	0.228	0.407	---	---	---
Instruction	0.186	0.003	0.061	0.008	0.087	0.094	0.001	---	---	---
T x I	0.063	0.016	---	0.002	0.019	0.026	0.006	---	---	---
Model 2: Total collapse (full)										
Task	0.500	0.782	---	0.263	0.285	0.240	0.152	---	0.514	---
Instruction	0.180	0.005	0.078	0.010	0.152	0.070	0.018	---	0.249	---
T x I	0.059	0.021	---	0.026	0.000	0.026	0.012	---	0.175	---
Model 3: Early adaptation (full)										
Task	0.480	0.790	---	0.591	0.209	0.253	0.454	---	---	0.018
Instruction	0.180	0.002	0.055	0.010	0.074	0.093	0.000	---	---	0.001
T x I	0.062	0.025	---	0.001	0.015	0.024	0.001	---	---	0.016
Model 4: Dynamic adaptation (full)										
Task	0.432	0.788	---	0.380	0.291	0.247	0.214	---	0.473	0.000
Instruction	0.151	0.005	0.070	0.009	0.130	0.083	0.014	---	0.187	0.007
T x I	0.054	0.022	---	0.036	0.001	0.025	0.010	---	0.141	0.003
Model 5: Cauchy-flight (full)										
Task	0.397	0.822	---	0.622	0.156	0.280	0.313	---	---	---
Instruction	0.175	0.006	0.057	0.036	0.069	0.097	0.000	---	---	---
T x I	0.077	0.021	---	0.003	0.002	0.039	0.000	---	---	---
Model 6: Lévy-flight (full)										
Task	0.441	0.806	---	0.605	0.168	0.300	0.419	0.074	---	---
Instruction	0.186	0.003	0.052	0.018	0.077	0.093	0.001	0.000	---	---
T x I	0.072	0.026	---	0.000	0.007	0.031	0.003	0.008	---	---

For the analyses of drift rates, we first entered the estimated drift rates from the single-stimulus condition and from the easy trials from the multi-stimulus condition in joint analyses. As could be expected, drift rates were notably smaller in the more difficult multi-stimulus task, all *Fs*(1,80)≥ 378.79; *ps*<.001, and effect sizes were comparable across models. In all simplified models without inter-trial variabilities (with exception of Model 1) there was also evidence for a small but significant increase of drift in the accuracy blocks compared to speed blocks. Interaction effects indicated that this effect of instructions was restricted to multi-stimulus tasks.

In a second set of analyses, drift rates from both easy and difficult trials of the multi-stimulus conditions were entered into separate 2 (instruction) × 2 (difficulty) repeated-measurement ANOVAs for each model. All models identified the difference in difficulty, all *Fs*(1,80) ≥ 387.09; *ps<*.001. Additionally, a main effect of instruction on drift was significant for all models except for the simple versions of Models 4 and 5. This effect again reflects faster evidence accumulation after speed than accuracy instructions.

Across all models, estimates for non-decision times were increased in the multi-stimulus task, all *Fs*(1,80) = 45.90; *ps<*.001. For the Cauchy Model (both in the simple and the complex version) and for the Lévy Model (only in the complex version), non-decision times were increased in the accuracy condition, all *Fs*(1,80) ≥ 6.02; *ps≤*.0.016.

Effects on the time of total collapse (λ) were estimated in Models 2 and 4. As expected, collapse of bounds was delayed in the multi-stimulus task, both *Fs*(1,80) ≥ 148.05; *ps<*.001, and following accuracy instructions, both *Fs*(1,80) ≥ 28.03; *ps≤*.001. Strong interaction effects show that the effect of instruction on λ is more pronounced in the multi-stimulus condition.

An early partial adaptation of boundaries (*δ*) was allowed in Models 3 and 4. Effects are weak and inconsistent: While the simple version of the early adaptation model (Model 3) suggests an early collapse especially for the easy single-stimulus task (*δ* < 0), *F*(1,80) ≥ 16.21; *p<*.001, an opposite pattern with the tendency for an early increase in threshold separation for the easy task is observed in the full version of this model, *F*(1,80) ≥ 4.96; *p=*.029.

Finally, the stability parameter (α) was estimated only in the Lévy-flight model (Model 6). A strong effect of task – observed both in the simple and full version of Model 6 – is based on higher stability of the noise distributions in the multi-stimulus blocks, both *Fs*(1,80) ≥ 28.87; *ps<*.001. Thus, the process is closer to standard diffusion in this condition than in the single-stimulus condition, where there seem to be larger jumps in evidence accumulation. Results from the simple version of Model 6 additionally suggest a decrease of alpha under speed instruction, *F*(1,80) ≥ 28.49; *p<*.001.

#### Model fit

In the assessment of model fit, the differences in complexity of the models need to be considered. For this purpose, model fit is assessed with both the AIC and the BIC, where BIC punishes model complexity more strongly than AIC. Information criteria were computed as shown in Eqs. 7 and 8, separately for each experimental condition, model and person (Voss et al., 2013):

7 $$ AIC=-2 LL+2\cdotp k, $$

8 $$ BIC=-2 LL+\ln (n)\cdotp k, $$

where LL is the log-likelihood, *n* is the number of trials (160 in the present case), and *k* is the number of estimated parameters (ranging from *k* = 3 for the simple version of Model 1 in the single-stimulus task to *k* = 9 for the full version of Model 4 in the multi-stimulus task). Mean AIC and BIC values are presented in Tables 5 and 6, respectively.

**Table 5:** Mean AIC values for all models

	Single-stimulus task		Multi-stimulus task	
	Speed	Accuracy	Speed	Accuracy
Simple Models				
1. Fixed Boundaries	1008	1019	1222	1398
2. Total Collapse	995	1014	1222	1407
3. Early Adaptation	992	1016	1218	1396
4. Dynamic Adaptation	993	1013	1221	1406
5. Cauchy Flight	995	1029	1236	1428
6. Lévy Flight	979	1003	1214	1395
Full Models				
1. Fixed Boundaries	977	1000	1212	1388
2. Total Collapse	980	1003	1249	1408
3. Early Adaptation	978	999	**1211**	1386
4. Dynamic Adaptation	980	1002	1237	1402
5. Cauchy Flight	979	1001	1220	1406
6. Lévy Flight	**975**	**998**	1212	**1385**

*Note:* Small values indicate good model fit. The best model for each condition (column) is printed in bold font

**Table 6:** Mean BIC values for all models

	Single-stimulus task		Multi-stimulus task	
	Speed	Accuracy	Speed	Accuracy
Simple models				
1. Fixed Boundaries	1,018	1,028	1,235	1,410
2. Total Collapse	1,007	1,027	1,237	1,422
3. Early Adaptation	1,004	1,028	1,233	1,411
4. Dynamic Adaptation	1,009	1,028	1,239	1,425
5. Cauchy Flight	1,004	1,038	1,248	1,440
6. Lévy Flight	**991**	**1,015**	**1,230**	1,410
Full models				
1. Fixed Boundaries	996	1,019	1,233	**1,409**
2. Total Collapse	1,002	1,025	1,273	1,433
3. Early Adaptation	999	1,021	1,236	1,411
4. Dynamic Adaptation	1,004	1,027	1,265	1,430
5. Cauchy Flight	997	1,019	1,242	1,427
6. Lévy Flight	997	1,020	1,237	1,410

*Note:* Small values indicate good model fit. The best model for each condition (column) is printed in bold font

According to the AIC, which incorporates only a moderate correction for model complexity, the full Lévy-flight model (i.e., Model 6, including inter-trial variability parameters) shows the best fit for the single-stimulus task under both instructions, and for the multi-stimulus task under accuracy instruction. For the multi-stimulus task under speed instructions, the fit of the full version of Model 3 (early adaptation) is slightly better than both standard diffusion (Model 1) and Lévy-flight (Model 6).

To avoid overfitting, it might be a good idea to punish model complexity more strongly by using the BIC to evaluate model fit (Table 6). Following this criterion, the simple versions of the Lévy-flight model show the best compromise of fit to data and parsimony for the easier single-stimulus task and for the speed instruction of the multi-stimulus task. Only for the multi-stimulus task under accuracy instructions does the full diffusion model show the best BIC value.

As a second strategy to assess fit of model predictions to data, we used a graphical approach. Appendix 2 shows the fits of model predictions to data for accuracy, and the 25%, 50%, and 75% RT quantiles for correct and error responses, for the diffusion model and the Lévy-Flight model. Generally, both models can predict the distributions of correct response times very accurately. For the error distributions, the diffusion model has some problems in predicting the leading edges in the fast conditions. We assume that the advantage of the Lévy flight model in this case is based on the capability of the Lévy-Flight model to account for fast errors. The pattern of results reverses for the slow experimental conditions: Here, a poorer performance of the Lévy Flight model might be explained with the absence of fast errors in data.

### Discussion

The present study aimed at comparing the ability of six variants of accumulator models to account for data from four variants of a number-letter classification task: In this paradigm, either one stimulus had to be categorized as a number or a letter (single-stimulus condition) or it had to be assessed whether the majority of 16 simultaneously presented moving stimuli were letters or numbers (multi-stimulus condition). Both conditions were completed under speed and under accuracy instructions.

The investigated models comprised: (1) the standard Wiener diffusion model, (2) a model assuming an eventual total collapse of boundaries, (3) a model with a fast early adaptation of boundaries, and (4) a model that combined an early adaptation of thresholds with a later total collapse. Next to the models assuming different shapes of decision boundaries, two further models were considered that used qualitatively different processes of information accumulation. These Lévy-flight models are based on heavy-tailed distributions for the noise of the information accumulation process. Unlike the diffusion model that uses a Gaussian noise distribution, a Cauchy distribution (Model 5) or a stable distribution (Model 6) were assumed for the Lévy-flight models. Both Gaussian and Cauchy distributions are special cases of the generic class of stable distributions that are characterized by values of α=2 and α=1, respectively, for the stability parameter. Lower values of the stability parameter denote a higher probability of extreme events: In the case of evidence accumulation, this means large random jumps in the information sampling. These six models were tested in a simple version (assuming constant parameter values across trials) and in a more complex version that allowed start point, drift, and non-decision time to vary from trial to trial of an experiment as is often done in diffusion modelling (Ratcliff & Rouder, 1998; Ratcliff & Tuerlinckx, 2002; but see Lerche & Voss, 2016, for a critical account on inter-trial variabilities).

#### The Wiener diffusion model

Model 1 is the standard diffusion model with constant boundaries: Data was collapsed over number and letter trials, thus estimating only one drift rate and no start point (relative start point ${z}_r=\frac{z}{a}$ was fixed at .5). For the simple and the full version of this model, three parameters (*a*, *v* and *t*_*0*_) or six parameters (additional parameters: *s*_*z*_, *s*_*v*_, and *s*_*t*0_), respectively, were estimated for each participant and condition. The simplified versions of all models served to prevent an over-parameterization for the more complex models discussed below. We showed elsewhere (Lerche & Voss, 2016) that validity and precision of estimates are often better when more restricted models are used.

Results were as expected: Threshold separations were higher under accuracy instructions compared to speed instructions (especially in the more difficult multi-stimulus condition). The drift rate was lower in the multi-stimulus condition than in the single-stimulus condition, which is plausible because of the much more difficult task. Threshold separation was increased for the more complex task. Obviously, participants compensate for the slower information sampling with a more conservative response style (Lerche & Voss, 2017). Only with such an increase in threshold separation participants can achieve a reasonable accuracy when information uptake is slow. Finally, in the multi-stimulus condition, non-decision processes take more time than in the single-stimulus condition. We assume that this finding is based on longer encoding of the multiple targets.

#### Models with adaptive thresholds

While the standard diffusion model assumes constant thresholds over time, it is plausible that decision-makers adapt their thresholds continuously. For example, they might reduce thresholds when at a certain point in time no sufficient amount of information has been accumulated. However, results from Model 2 (total collapse) indicate that a collapsing of boundaries has only a small impact on the performance in the present tasks. The estimate for the time of collapse is so late that changes in thresholds are minimal for the majority of observed responses. Consequently, results for the other model parameters mimic results from Model 1.

Models 3 and 4 allow for an early partial adaptation of thresholds. On average, these early adaptations are rather small (with a maximum of 10% collapse), and results for the remaining parameters are not influenced substantially.

#### Lévy-flight Models

In Models 5 and 6, we assumed that the noise of information accumulation is described by a Cauchy distribution, or by the more generic alpha-stable distribution, respectively. Both distributions have heavy tails, which means that they allow for an increased probability of extreme events, that is, of large jumps in the information accumulation process. Because model fit is rather poor for the Cauchy model, we will not discuss results from Model 5. In Model 6, the stability parameter α of the stable distribution was estimated as an additional free parameter in the model. This allows mapping a contingency between a standard diffusion model with normal noise (α=2) and a Lévy model with Cauchy noise with many extreme jumps (α=1). Results reveal a strong effect of task on stability: In the multi-stimulus condition, α is notably larger compared to the single-stimulus condition, suggesting that for the latter a standard diffusion model is less appropriate. This is plausible, because in the single-stimulus condition, continuous accumulation of information is less likely than in a multi-stimulus task, where different sources of information need to be integrated. Since many of the standard diffusion model tasks are based on very easy classifications, this finding strongly suggests Lévy-Flight models should be applied more often.

#### Model fit

The present study allows only a preliminary assessment of model fit for two reasons: Firstly, trial numbers (160 trials per condition) are rather low, which makes a precise estimation of model fit difficult. Secondly, there is the problem that the likelihood function is unknown for some of our models, and thus likelihood had to be estimated from quantile probability estimation, which enters additional inaccuracies both in parameter estimation and in the assessment of fit. Nonetheless, our results allow for a first comparison of the capability of the different models to account for our data. Since any evaluation of the fit of a mathematical model should not only consider how well data are recovered but also address model complexity, we assessed model fit with the information criteria AIC and BIC.^9^ This allows us to test which model shows the optimal compromise of fit and parsimony for each experimental condition.

In accordance with previous results (Hawkins et al., 2015; Voskuilen, Ratcliff, and Smith, 2016), a model assuming a total collapse of thresholds (Model 2) did not substantially increase model fit compared to the standard diffusion model (Model 1). However, fit for the model that allowed for a partial adaptation of thresholds (Model 3) was generally better than for the diffusion model. Interestingly, this increased fit is not due to a general decline of threshold separation. Rather, our results suggest substantial inter-individual differences, with some participants decreasing, some increasing threshold separation. Assuming a two-step adaptation of thresholds (Model 4) did not bring a substantial additional advantage for model fit.

Models with a Cauchy-distributed noise (Model 5) performed better than standard diffusion for the condition with fastest responses (single-stimulus task under speed instructions), but showed a worse fit in all other conditions. The Lévy-flight model that estimated stability parameter alpha as a free parameter (Model 6) generally had a better fit than standard diffusion for the simple version of models (excluding inter-trial variabilities). In fact, when the BIC is considered – which more strongly favors parsimonious models – the simple version of the Model 6 outperformed all other models (including the much more flexible full versions) for three of the four experimental conditions; only in the slowest condition did the model assuming early adaptation outperform the Lévy-flight model. Obviously, the advantage of the Lévy-flight model over standard diffusion is especially strong for the single-stimulus task. This is true for both instructions, for full and simple models, and regardless of whether AIC or BIC is considered.

To improve understanding why Lévy-flight models account better for our data than the standard diffusion model, we investigate how predicted RT-distributions differ between both classes of models in the next section of this paper.

## Predictions from Lévy-flight models: Fast errors and reduced skew

**Fig. 2:**
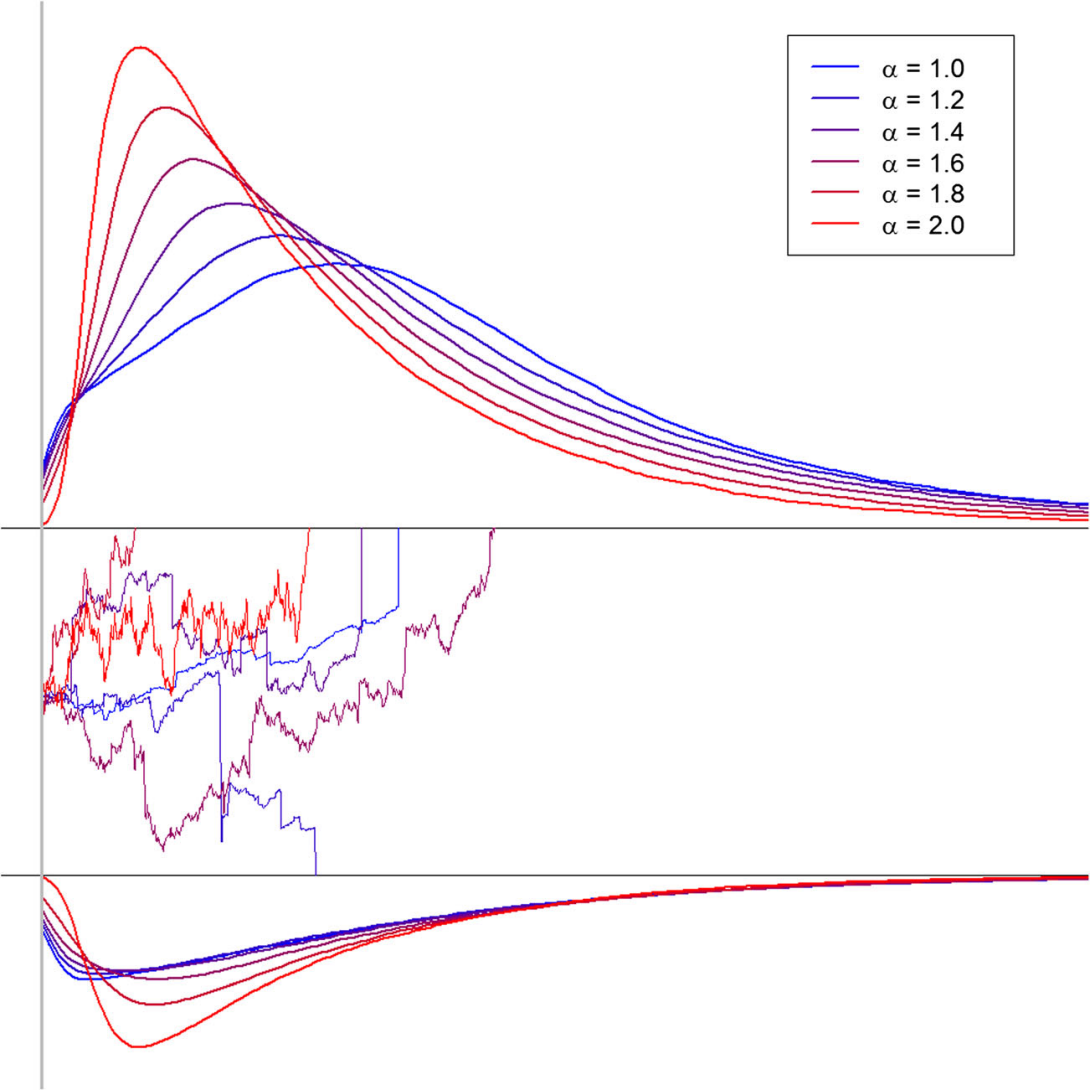
Predicted decision-time distributions for correct responses (upper threshold) and error responses (lower threshold) from accumulator models with six different alpha-stable noise distributions. Further explanations are given in the text

To assess the reasons for the good fit of the Lévy-flight model, we analyzed the characteristics of the predictions of this model class. For this purpose, 10^6^ process paths were simulated for each of six values of the stability-parameter alpha (1.0, 1.2, 1.4, 1.6, 1.8, 2.0), covering the complete range from a Cauchy distribution to a Gaussian distribution for the noise. For these simulations, constant thresholds with *a* = 2, a centered start point (*z*_*r*_ = .5), and a small positive drift rate (*v* = 1) were used. Resulting decision times are depicted in Table 7 and Fig. 2.

**Table 7:** Predictions for decisions and decision times of the Lévy-flight models as a function of the stability parameter α

α	pc	DT correct			DT error		
		*M*	*SD*	Skew	*M*	*SD*	Skew
1.0	.78	753	537	1.60	556	519	1.90
1.2	.79	721	536	1.72	569	530	1.92
1.4	.78	673	517	1.81	571	516	1.87
1.6	.78	617	483	1.86	561	488	1.91
1.8	.76	553	439	1.92	532	442	1.88
2.0	.74	486	389	1.96	486	389	1.95

*Notes*: DT=Decision time (in ms); to get response times, a non-decision time has to be added. *pc*=percent correct. For the simulations, the following parameter values were used: *a*=2, *z*_*r*_=.5, *v*=1

The most striking result of this simulation is the finding that the Lévy-flight model predicts fast errors, with the difference between correct decision latencies and error response latencies increasing with decreasing stability parameter. While in the diffusion model (α=2) errors and correct responses do not differ in speed, for the Cauchy noise (α=1), the erroneous decisions are on average almost 200 ms faster than correct decisions. This pattern emerges, because – in the Lévy-flight mode – errors are typically based on large jumps in the “wrong” direction, which can already occur within the first milliseconds of the decision-process. Correct responses, on the other hand, emerge predominantly in trials without any larger jumps; in this case, the constant drift brings the process eventually to the correct threshold. Because this takes longer, mean RTs are slower for correct responses. This characteristic of Lévy-flight models provides a completely new explanation for fast error responses.

Another characteristic of the predicted decision-time distributions are the very steep lower edges of the distributions, which are again based on the fact that jumps in the decision process can lead to decisions within the first few milliseconds. Additionally, standard deviations are increased, and skew for correct responses is decreased with lower stability of the noise distribution (see Table 7).

## General discussion

In the present paper, we compare the ability of 12 different variants of accumulator models to account for data from a number-letter classification task. Specifically, we compared performance of the standard diffusion model with models that allow for dynamic adaptations of threshold separation within the decision process. Additionally, two further models assumed constant thresholds but a different accumulator process. Whereas in the diffusion model random fluctuations of the decision process are introduced by adding Gaussian noise to a constant drift, the new models assume more heavy-tailed noise distributions, thus allowing for larger jumps in the evidence accumulation process. For these noise distributions, so-called alpha-stable distributions were used. The resulting processes that are used to map the decision process are called Lévy-flights. Six different models were tested in a parsimonious version assuming constant parameter values across trials, and in a full version, allowing for inter-trial variability of start point, speed of evidence accumulation, and non-decision times (Ratcliff & Rouder, 1998; Ratcliff & Tuerlinckx, 2002).

### Adaptation of thresholds

One reason to introduce collapsing boundaries in an accumulator model is that the collapse of bounds sets an upper limit for response times. When the accumulated information does not suffice to make an informed decision (based on the *a priori* set criteria) after a certain time, decision-makers may become impatient and adapt their decision style by lowering these criteria. However, previous research found little evidence for the impact of collapsing boundaries in humans (Hawkins et al., 2015; Voskuilen, Ratcliff, and Smith, 2016). Replicating these findings, the collapse observed in our studies was rather small as well: Models assuming a total collapse (Model 2) suggest that thresholds remain close to their initial values for quite a while, and models incorporating early collapse (Model 3) show that these early adaptations are rather small on average (although there was substantial variability across participants).

Finally, we combined the ideas of early adaptation and later total collapse: Model 4 allowed for an early partial adaptation and a later total collapse. Results mimic those of Model 3, that is, we again find little evidence of a general adaption but a rather large variance, suggesting that some participants increase and some decrease threshold separation initially.

### Lévy-flight models

In addition to the adaptation of boundaries, the present study also analyzes the impact of heavy-tailed noise distributions in the accumulation process. Heavy-tailed distributions have an increased probability of extreme events. In the case of evidence accumulation, such extreme events reflect large sudden changes in accumulated evidence, which could – psychologically – indicate a sudden insight of the decision-maker. If such a sudden jump in accumulated evidence directly reaches the threshold, this resembles a phenomenon dubbed as “jumping to conclusion” (McKay, Langdon, & Coltheart, 2006). Importantly, in our modelling approach jumps in both directions have the same probability, that is, they can support correct or erroneous decisions. In Model 5, we used the Cauchy distribution to model random influences in the decision process. This model, having the same degrees of freedom as the simplified diffusion model, fitted data well for the single stimulus condition under speed instructions. Generally, however, Model 6, estimating different stability values for all participants (thus adding one degree of freedom), shows an even better model fit. When fit is evaluated with the BIC, which strongly punishes model complexity, a simple version of the Lévy-flight model outperforms the full-diffusion model in all but the slowest condition (i.e., multi-stimulus-task with accuracy conditions).

In the family of Lévy stable distributions, the stability parameter alpha has a possible range of 0–2, where *α* = 0.5, *α* = 1 and *α* = 2, results in Lévy-, Cauchy, and normal distributions, respectively. We find mean alpha values around 1.5 for the easy single-stimulus task and around 1.8 for the more difficult multi-stimulus task, indicating that deviations from predictions of the standard diffusion model are notably stronger for the former task. This corresponds to the fact that for easy tasks, especially under speed instructions, error responses are typically faster than correct responses (Luce, 1986). This pattern is predicted by accumulator models assuming heavy-tailed noise. We hypothesize that the Lévy-flights model the mechanism underlying this effect.

In the diffusion model context, fast errors have been previously explained by inter-trial variability of the start point (Ratcliff & Rouder, 1998; Smith, Ratcliff, & Sewell, 2014). For a theory of decision-making, it is an important research question to differentiate empirically between both approaches. Our data shows a better fit for the Lévy-flight models. However, this finding should be replicated with notably larger data sets.

The observed differences of the stability parameter alpha might help to gain some insights in the psychological processes mapped by this parameter. We assume that normal noise in evidence accumulation arises, when multiple sources of information (e.g., multiple stimuli) have (nearly) simultaneous impact on the decision process. In this case, conflicting pieces of evidence cancel out each other and so foster a moderate speed of evidence at most points in time. It is plausible that evidence accumulation is less smooth, when attention is focused only on one stimulus, and the identification of a simple perceptual feature that might strongly suggest one response is not counterbalanced by other information.

Furthermore, we hypothesize that a Lévy-flight could reflect particular efficient decision-making in certain situations. Our argument is related to an account from biology, denoted as the Lévy-flight foraging hypothesis (Viswanathan, Raposo, & Da Luz, 2008). This hypothesis assumes that movements of a two-dimensional Lévy flight can maximize foraging results for animals (e.g., for albatrosses; see Reynolds, 2012). In decision-making, a flexible, rapid switching between the testing of different perceptual hypotheses (e.g., possible targets) could lead to large fluctuations in the decision process. The idea that a low stability reflects efficient decision-making is supported by some very preliminary (and unpublished) findings from further studies from our lab, suggesting that alpha is negatively related to intelligence and positively related to impulsivity and negative emotionality of borderline patients. Expanding this argument further, a highly stable diffusion process would reflect an inefficient way of decision-making, for example, suggesting that a decision-maker is not able to switch attention rapidly between different possible target concepts.

The present paper does not focus on the question of inter-individual differences in the noise-distributions of the accumulation process; however, we argue that it is plausible to assume meaningful variance here. The individually estimated α-values (in all four conditions of the present experiment) show considerable standard deviations: For some persons, a standard diffusion model describes evidence accumulation accurately. For others, the process is closer to a Lévy-flight with Cauchy-distributed noise. Since the alpha parameter (in contrast to inter-trial variability of start-point or drift) can be estimated reliably (see Appendix 1), we believe it would be a fruitful endeavor to further investigate the relation of this parameter to measures like cognitive ability (e.g., executive functions), personality (e.g., impulsivity), or clinical symptoms (of, e.g., Borderline patients).

### Some methodological caveats

We conclude this paper by stressing some methodological caveats that require further research. While adaptive boundary models have been investigated repeatedly before, the present study is to the best of our knowledge the first study using Lévy-flights to model decision-making. Therefore, more research is necessary to confirm our results. From a methodological perspective, several limitations need to be considered carefully.

Firstly, the trial numbers used in the present study are too low to allow an exact evaluation of model fit. Larger trial numbers are necessary not only to make parameter estimation more stable, but also to make a more meaningful investigation of model fit possible.

Secondly, the applied method of parameter estimation based on the quantile maximum probability estimation (Heathcote, Brown, & Mewhort, 2002) is not optimal. Alternative approaches should be developed that allow an accurate recovery of parameters in a reasonable time frame. One possible approach that might succeed here is the probability density approximation (PDA; Holmes, 2015). However, the PDA approach might be even more time-consuming than the here-applied SIMPLEX search. Other approaches that might allow for a faster parameter estimation comprise Approximate Bayesian Computation (Mertens, Voss, & Radev, 2018) or machine-learning algorithms based on Deep Inference (Radev, Mertens, Voss, & Köthe, 2018). Future research is necessary to assess which methods are least biased and most efficient.

Thirdly, it is necessary to validate the stability parameter alpha in psychological terms, that is, it should be investigated, what – if anything – is measured by α. Right now, the idea that jumps in the accumulation processes represent an effective switching of attentional resources is rather speculative.

## Figures and Tables

**Fig. 3:**
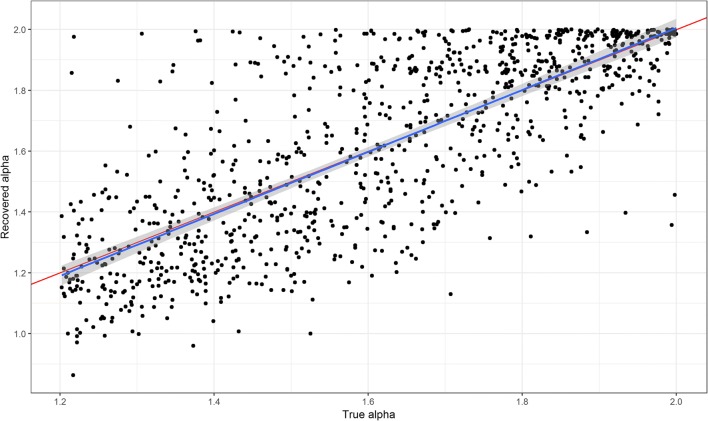
Recovered values for the stability parameter alpha as function of the true values entered into the simulation. The blue line shows the linear regression of recovered values to true values, with the grey area giving the 99% confidence interval. The red line shows perfect relation

**Fig. 4:**
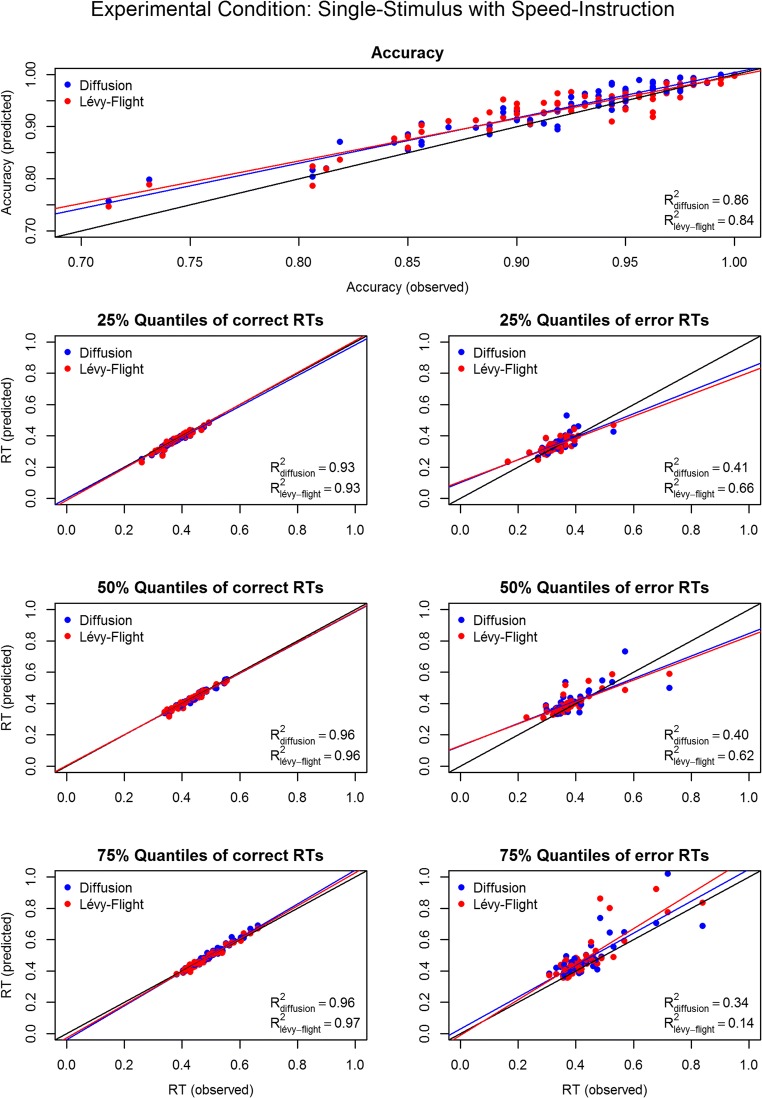
Correspondence of model predictions for accuracy and response time quantiles for the single stimulus task under speed instructions

**Fig. 5:**
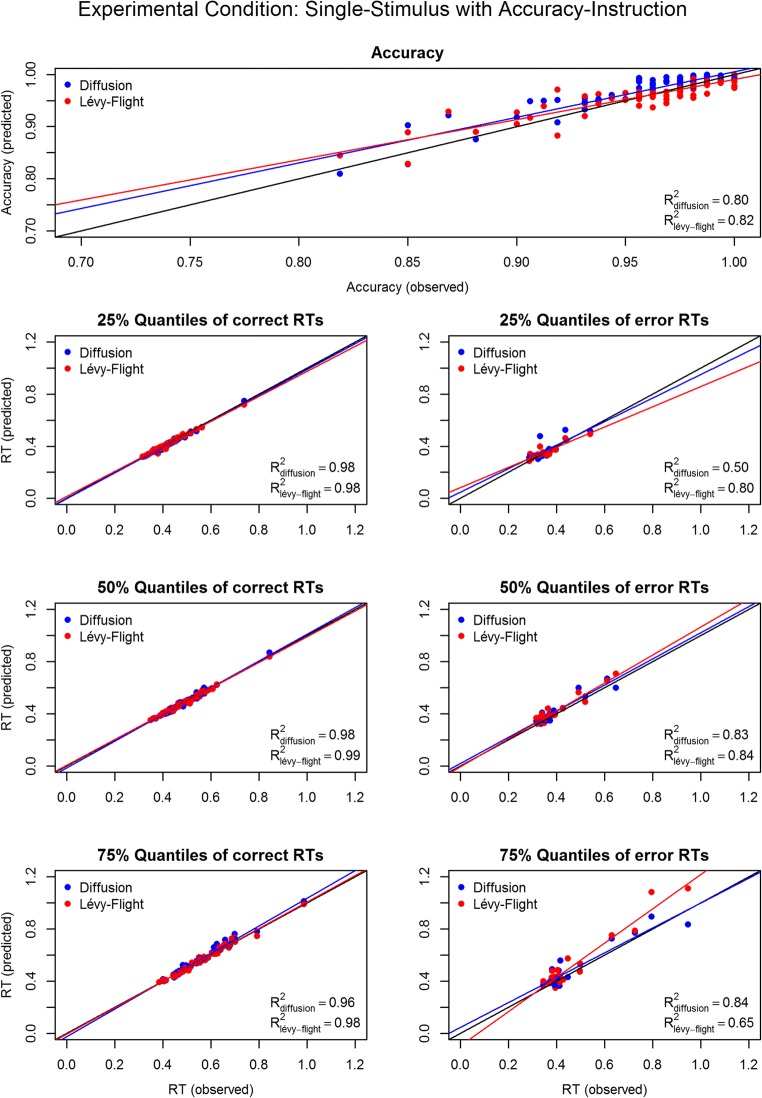
Correspondence of model predictions for accuracy and response time quantiles for the single stimulus task under accuracy instructions

**Fig. 6:**
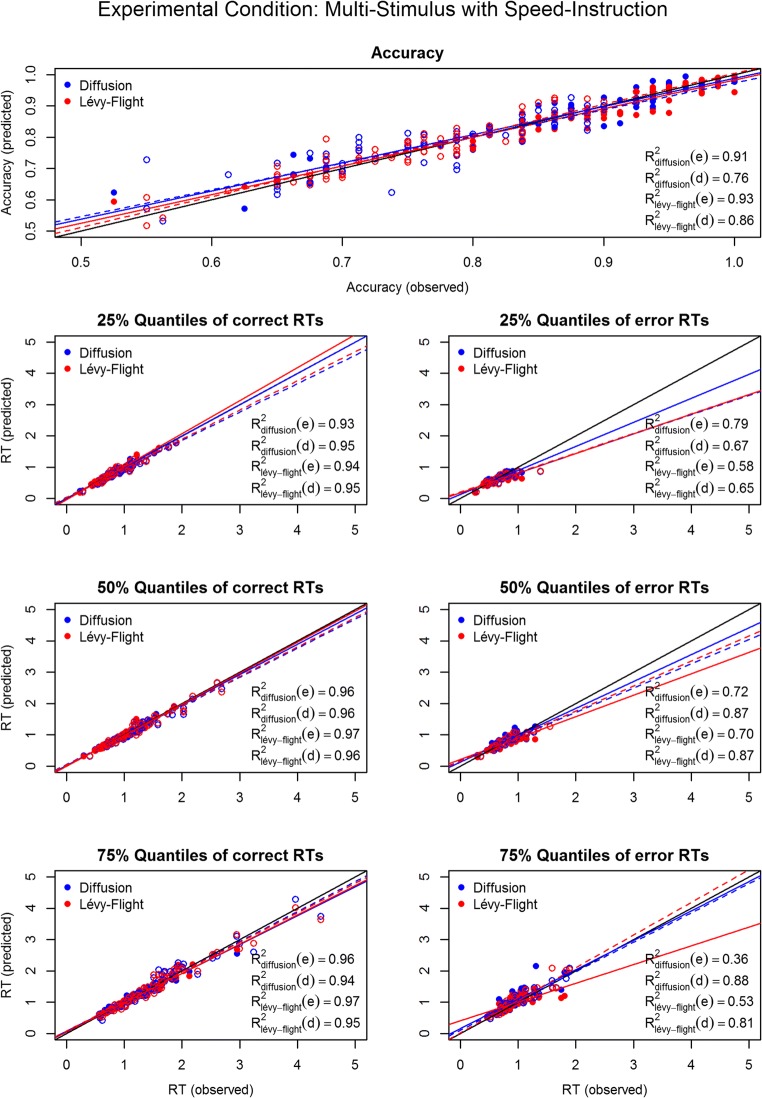
Correspondence of model predictions for accuracy and response-time quantiles for the multi-stimulus task under speed instructions. Solid circles and solid lines represent the easy trials (e); open circles and dashed lines represent the difficult trials (d)

**Fig. 7:**
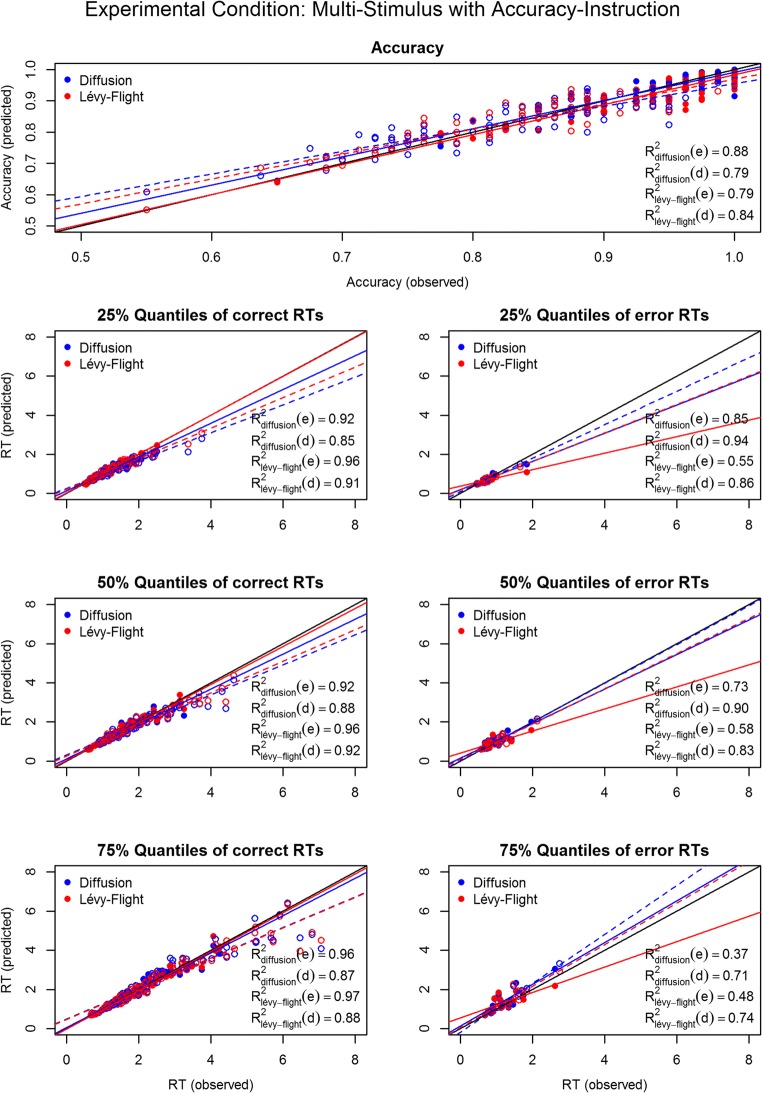
Correspondence of model predictions for accuracy and response time quantiles for the multi-stimulus task under accuracy instructions. Solid circles and solid lines represent the easy trials (e); open circles and dashed lines represent the difficult trials (d)

## Appendix A: Parameter recovery

With this simulation study, we demonstrate the quality of parameter recovery using our implementation of the quantile maximum probability estimation (Brown & Heathcote, 2003; Heathcote & Brown, 2004) for the newly developed Lévy-flight model (Model 6).

### Method

One thousand parameter sets were drawn from independent uniform distributions that correspond roughly to the ranges for the model parameters estimated from data of the single-stimulus condition (Table 8). Then, responses from 160 trials were simulated from each parameter set, and parameters were re-estimated following the procedure described in the main part of the paper.

**Table 8.** Ranges for the different parameters used in the simulation

Parameter	Minimum	Maximum
Threshold (*a*)	0.75	2.00
Drift (*v*)	1.50	5.00
Non-decision-time (*t*_0_)	0.20	0.30
Stability Parameter (*α*)	1.20	2.00

### Results

As Table 9 shows, strong correlations of estimated values with true values emerged for all parameters. For stability parameter alpha, we additionally show the fit in Fig. 3. As can be seen, the diagonal, representing perfect recovery, falls within the confidence intervals for the regression of recovered values to true values, suggesting that estimates are unbiased.

**Table 9.** Correlations of true parameter values with recovered values

	recovered			
true	*a*	*v*	*t*_0_	*α*
*a*	**.70**	-.23	.08	-.13
*v*	-.30	**.89**	.23	-.14
*t*_0_	-.08	.02	**.73**	-.03
*α*	-.13	-.04	.14	**.77**

## Appendix B: Recovery of accuracy and response time quantiles

To assess model fit graphically, accuracy and the 25%, 50%, and 75% response time quantiles for correct responses and error responses were predicted from estimated parameter values of all participants and all experimental conditions, using the full versions of the standard diffusion model (Model 1) and of the Lévy-flight model (Model 6). Predictions are based on 2000 simulated responses for each condition. These predictions are compared to data in Figs. 4, 5, 6, and 7. For quantiles of error responses, only data from participants making at least eight errors in the respective condition are shown, because otherwise observed RT quantiles lack the required precision.

The figures generally indicate a good to very good prediction for correct responses, and poorer fit for error responses. This can be expected, on the one hand, because errors are rare and thus have not as strong an influence on parameter estimation as correct responses. On the other hand, quantiles of observed responses are measured less precisely, because only very few errors occur (especially in the single-speed task under accuracy instructions). For the single stimulus task, the Lévy-flight model has slightly superior fit for the first quartile of error responses, as indicated by higher *R*^2^ values. This might be the reason for the overall better fit of the Lévy-Flight model in this condition.

## Footnotes

1 Note that even for *v*=0 thresholds will be reached eventually, because of the random noise. In this case, probabilities of hits at the upper and lower thresholds are identical (*p* = .5).

2 Lévy-flights form a superordinate model class that comprise both models with heavy-tailed and with normal-noise distributions. However, in this paper we use the term Lévy-flight in contrast to a standard diffusion model to denote models with heavy-tailed noise.

3 Although we denote Model 6 as Lévy-flight, it is important to mention that we implemented one special sub-form of the broad class of mathematical Lévy-flights here, that is, a process with constant drift and alpha-stable noise. Further information on the general class of Lévy-flights can be found, for example, in Kallenberg (2006).

4 The standard deviation of the noise is called the diffusion constant and it operates as a scaling parameter. Some applications of the diffusion model are based on a diffusion constant of *s* = 0.1. To make results from the present paper comparable to results from papers using other diffusion constants, parameters *a*, *v*, and *z* (and the corresponding inter-trial variabilities) have to be rescaled by multiplication with the new diffusion constant.

5 http://www.gnu.org/software/gsl/

6 Movements were always straight with a random direction. Horizontal and vertical speeds were randomly determined with a maximum speed of 256 pixels per second. When the edge of the screen was reached, stimuli bounced back in a new random direction.

7 Twelve participants had to be excluded from this ANOVA because they made no error in at least one condition. An analysis of correct response times for all participants leads to very similar results.

8 Because the models differ notably, it is unclear whether parameters are directly comparable regarding their means and variances. Thus, we decided not to enter model as a third factor and describe the congruency of results in a descriptive way.

9 Future research on model fit might measure model complexity not only by the number of free parameters, but could also consider the functional form of the models: Assuming different noise distributions might increase model complexity more than assuming a gradual collapse of decision thresholds.
